# The Neural Correlates Underlying Belief Reasoning for Self and for Others: Evidence from ERPs

**DOI:** 10.3389/fpsyg.2016.01501

**Published:** 2016-10-04

**Authors:** Qin Jiang, Qi Wang, Peng Li, Hong Li

**Affiliations:** ^1^School of Education, Guangxi University, NanningChina; ^2^Institute of Education Sciences, Chengdu University, ChengduChina; ^3^Department of Psychology, Sun Yat-sen University, GuangzhouChina; ^4^College of Psychology and Sociology, Shenzhen University, ShenzhenChina

**Keywords:** belief reasoning, theory of mind, self, others, decoupling, late positive component

## Abstract

Belief reasoning is typical mental state reasoning in theory of mind (ToM). Although previous studies have explored the neural bases of belief reasoning, the neural correlates of belief reasoning for self and for others are rarely addressed. The decoupling mechanism of distinguishing the mental state of others from one’s own is essential for ToM processing. To address the electrophysiological bases underlying the decoupling mechanism, the present event-related potential study compared the time course of neural activities associated with belief reasoning for self and for others when the belief belonging to self was consistent or inconsistent with others. Results showed that during a 450–600 ms period, belief reasoning for self elicited a larger late positive component (LPC) than for others when beliefs were inconsistent with each other. The LPC divergence is assumed to reflect the categorization of agencies in ToM processes.

## Introduction

In everyday life, people need to think about the mental states of both themseleves and others, and then understand and predict behaviors based on that knowledge. This ablility, playing a core role in social interaction, is called the theory of mind (ToM) in psychological research. Since Premack and Woodruff (1978) proposed the notion of ToM, many studies have been conducted to understand the developmental processes in ToM, and how these processes are affected by particular psychological or environmental factors (Wellman, 1992). However, we know very little about the cognitive mechanisms of how the processing of ToM takes place in the brain as yet. In recent years, the extensive use of event-related potential (ERP) methodology provides opportunities for researchers to study this question from a cognitive neuroscience perspective, profiting from its high temporal resolution.

Many studies suggest that ToM includes an understanding of different types of mental states, such as: desire, belief, intention, and emotion (Wellman, 2011). To date, belief has attracted the most abundant interest in research as a typical mental state. The acquisition of belief reasoning is considered to be a milestone in ToM development. The electrophysiological mechanisms involved in belief reasoning have been explored by using the ERP methodology. In the matter of paradigm, prior work can be approximately divided into three categories.

The first type of research focuses on comparing the neural activity elicited by belief to that by non-mental representations. For instance, Sabbagh and Taylor (2000) asked participants to reason about belief representations and photograph representations. They found that these two kinds of reasoning decoupled on a slow late component over left frontal areas. Liu et al. (2004, 2009b) compared ERPs elicited by judgments based on belief to those based on reality for adults and children. Results also confirmed that belief reasoning was associated with a late slow wave (LSW) with a left frontal scalp distribution.

The second kind of research mainly concerns the ERP differences between belief reasoning and other types of mental state, such as desire reasoning, pretense, and so on. Liu et al. (2009a) investigated the neural correlates of belief reasoning and desire reasoning in adults and children (Bowman et al., 2012). Results revealed that both belief and desire reasoning were associated with an anterior LSW. Studies explored the neural basis of belief reasoning and pretense indicated that false belief reasoning evoked an anterior LSW (600–900 ms) for adults and a fronto-cental LSW (290–920 ms) for children, which are assumed to reflect the decoupling mechanism of metarepresentation (Meinhardt et al., 2012; Kühn-Popp et al., 2013).

The third type of research focuses on ERPs that distinguish between different kinds of belief reasoning. For example, Meinhardt et al. (2011) found that there were two ERP components differentiating false from true belief reasoning, including a late positive component (LPC, 300–600 ms) and a late anterior slow wave (LSW, 600–900 ms). Geangu et al. (2013) recorded ERPs of participants who passively viewed photographic sequences of a character performing actions under either false or true belief conditions. The results are similar to those from studies asking participants to conduct belief reasoning directly (e.g., Meinhardt et al., 2011). A frontal LSW within the 570–690 ms epoch was associated with the processing of false belief. Another ERP study compared the brain activities elicited by the standard false belief reasoning task to that by the adapted false belief reasoning task. In the standard task, participants could see that an object was transferred from location A to location B, while in the adapted task, an object was transferred from location A to a place that participants did not know. It was proposed that the adapted task had lower inhibiting demands since participants had no need to inhibit their knowledge about the real location of the object. The standard task elicited a more positive LPC at 470–520 ms than that in the adapted task (Zhang et al., 2009).

Taken together, there is a general agreement that ERP components associated with belief reasoning, such as LPC and LSW, are normally later than 300 ms after the onset of stimulus. However, the meaning of these components in belief reasoning is controversial due to the variety of paradigms used in existing studies. For example, previous studies suggest that LPC is associated with the process of inhibition involved in belief reasoning (Zhang et al., 2009), or the reorientation from external stimuli to internal mental representations (Meinhardt et al., 2011). For LSW, it has been proposed that it reflects the processes associated with distinguishing mental states from reality (Sabbagh and Taylor, 2000; Liu et al., 2004), or the decoupling mechanism involved in metarepresentation (Meinhardt et al., 2012; Kühn-Popp et al., 2013). Consequently, it is necessary to examine the neural activities in different forms of belief reasoning tasks, which might provide more information about the cognitive mechanisms involved in ToM processes.

Although the definition of ToM contains the understanding of mental states for both self and others, previous studies mainly focus on mental reasoning for others, usually a protagonist in scenarios presents with text or photographs. Actually, in the ToM tasks asking participant to reason about the mental state of other people, the mental state of participant’s own must also be taken into account. The decoupling mechanism of processing the mental state of others independently from our own mental state constitutes a core component of ToM (Gallagher and Frith, 2003).

Neuroimaging studies concerning mental reasoning for different agencies (self or others) have been somewhat limited. Vogeley et al. (2001) investigated the neural mechanisms of taking self-perspective and modeling the mind of someone else in a ToM paradigm. Results showed that both taking self-perspective and modeling the mind of someone else led to increased neural activity in the right prefrontal cortex. Saxe et al. (2006) compared the neural activities of belief reasoning for others and trait adjective judgment for self. They found that both tasks activated the medial prefrontal and medial precuneus significantly. A recent meta-analysis revealed that both self and other judgments are associated with activity in the medial prefrontal cortex (MPFC). Specifically, self-related judgments are more frequently activated the ventral MPFC, whilst other-related judgments more frequently activate the dorsal MPFC (Denny et al., 2012). In addition, there is some evidence that sub-regions of the MPFC distinguish between thinking about similar and dissimilar others. The dorsal MPFC is recruited for reasoning about dissimilar others, whilst the ventral MPFC is used in reasoning about self or similar others (Mitchell et al., 2005, 2006; Pfeifer et al., 2007). These findings suggest that belief reasoning for different agencies may at least partly implement distinct neural bases.

Moreover, there are two recent studies specifically investigated the neural underpinnings of the decoupling mechanism distinguishing mental states belonging to different agencies in ToM reasoning. In an fMRI study conducted by Schuwerk et al. (2014a), the results suggest that the posterior medial prefrontal cortex (pMPFC) helps to process others’ inconsistent beliefs decoupled from one’s own perspective. Furthermore, Schuwerk et al. (2014b) modulated pMPFC activity by repetitive transcranial magnetic stimulation (rTMS) and observed that the inhibition of this region impaired the ability to distinguish the other’s belief from one’s own. These findings support the important role of pMPFC in the decoupling mechanism, however, the time course of the process to separate and compare the mental states of different agencies (self or others) remains unclear.

The present study aimed to investigate those ERP components associated with the decoupling mechanism in ToM reasoning by comparing the neural correlates underlying belief reasoning for self and for others. Furthermore, the consistency of beliefs between one’s own and that of others was also considered. In consistent conditions, participants had the same beliefs with a protagonist in ToM scenarios, whilst in inconsistent conditions they held conflicting beliefs to the protagonist. Given the core role of ToM in interpersonal interaction, the present study allows us to gain an insight into the cognitive neural mechanism of how the ToM processed in social interaction between self and others. It also helps us to understand better some social developmental disorders such as autism which is characterized by deficits in interpersonal interaction.

## Materials and Methods

### Participants

Seventeen participants voluntarily took part in the present task. All of them were right handed and with normal or corrected to normal vision. A participant was excluded because of a failure of the electricity supply during the experimental procedure. The remaining sixteen participants (seven males) were aged between 19 and 24 years (*M* = 21.28 years, *SD* = 1.5 years). All participants received monetary compensation for their participation and signed an informed consent for the experiment before the testing. The experiment was approved by the Academic Ethics Committee of Guangxi University.

### Procedure

The present belief reasoning task is based on the diverse-beliefs task, which is a classic ToM test and is used in several developmental and ERP studies (Wellman and Woolley, 1990; Wellman and Liu, 2004; Wellman et al., 2006; Liu et al., 2009a; Bowman et al., 2012). For example, in a typical diverse-beliefs task, participants were told that there were two different positions in which a cat might be hidden (e.g., the bushes and the garage) and they were asked where they thought the cat was. After participants answered (e.g., the bushes), they were told that a character, Linda, thought that the cat was in the other position (e.g., the garage). Then, participants were asked to predict where Linda would look for the cat (the bushes or the garage). The present belief reasoning task was a modified version of the typical diverse-beliefs task. A 2 (agency: self vs. other) × 2 (consistency: consistent vs. inconsistent) design required all participants to complete four experimental conditions, including self-consistent, self-inconsistent, other-consistent, and other-inconsistent conditions.

**FIGURE 1:**
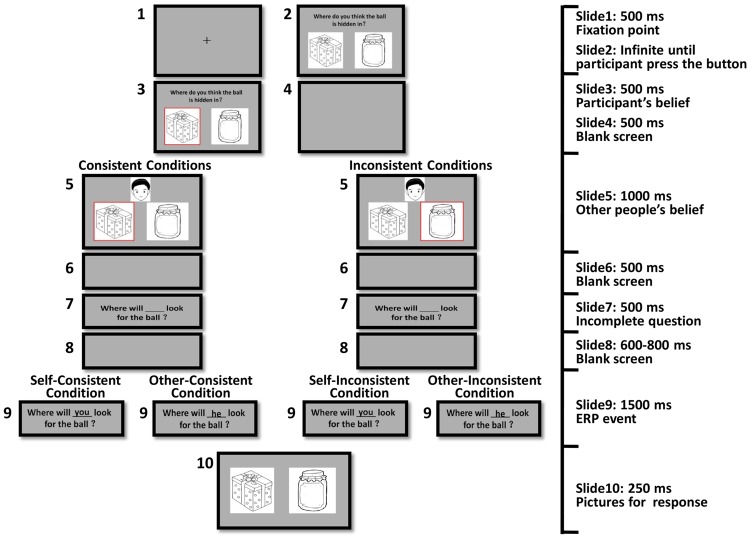
**Experimental procedure**.

The structure of the four conditions was similar. Participants were presented with a picture of a little ball and two pictures of containers (e.g., a box and a jar). Then the ball disappeared and participants were asked to choose the container in which they thought the ball was hidden. Participants pressed “F” if they thought the ball was hidden in the left container or “J” for the right container. After they pressed a key, a red frame would surround their chosen container (e.g., the box). A headshot of a boy/girl (each for half of the trials) was then presented with a red frame surrounding one of the two containers, meaning that the boy/girl thought the ball was hidden there in. For consistent conditions, the boy/girl had the same belief about where the ball was as the participants (e.g., the ball was hidden in the box). For inconsistent conditions, the boy/girl had a different belief about where the ball was with the participants (e.g., the ball was hidden in the jar). Next, participants read an incomplete question: “Where will ___ look for the ball?” After a blank screen, the question was completed by a word of agency presented in the blank space. For self conditions, the word would be “you,” asking participants to predict their own behavior. For other conditions, the word would be “he/she,” asking participants to predict the boy/girl’s behavior. The presentation of the completed question (“Where will you look for the ball?” or “Where will he/she look for the ball?”) was the target event for ERP analysis. Participants were presented with the complete question for 1500 ms then the two pictures of the containers were presented again for a further 250 ms. Participants needed to provide their answers by pressing “F” for the left container and “J” for the right container. When the pictures of the two containers were presented again, their locations were the same as when they were shown at the first time in half of the trials and reversed in the other half. **Figure 1** illustrates the procedure for the four experimental conditions when the other headshot showed a boy.

There were 72 trials for each condition in the formal experiment. Two hundred eighty-eight trials in total were randomly presented in four blocks. Participants began the formal experiment only if they could give the correct response in 10 consecutive training trials, which ensured that they fully understood the structure of the task.

### Electrophysiological Recording and Analysis

The electroencephalogram (EEG) was recorded from BrainAmp amplifiers (Brain Product, Herrsching, Germany) with 64 Ag/AgCl electrodes mounted on an elastic cap according to the International 10–20 system. An electrode below the right eye was used to monitor the vertical electrooculogram (EOG) and another electrode at the external outer canthi of the left eye was used to monitor the horizontal EOG. The reference electrode was positioned at FCz and the ground electrode was positioned at AFz. All electrode impedances were maintained below 5 kΩ. The EEG and EOG were acquired with a sampling rate of 500 Hz, a band pass of 0.01–100 Hz. In off-line analysis, electrodes were re-referenced to average mastoids and a digital band pass filter of 0.01–30 Hz was applied. EOG artifacts from eye movement were corrected using an independent component analysis algorithm. The segment epoch for ERP was 1200 ms, including a 200 ms pre-stimulus baseline. Segments with an incorrect response or a peak-to-peak deflection exceeding ±100 μV were excluded from the final averaging. As a result, the average number of trials per condition submitted for final analysis was 68 for the self-consistent, 67 for the self-inconsistent, 68 for the other-consistent, and 62 for the other-inconsistent conditions, respectively.

**FIGURE 2:**
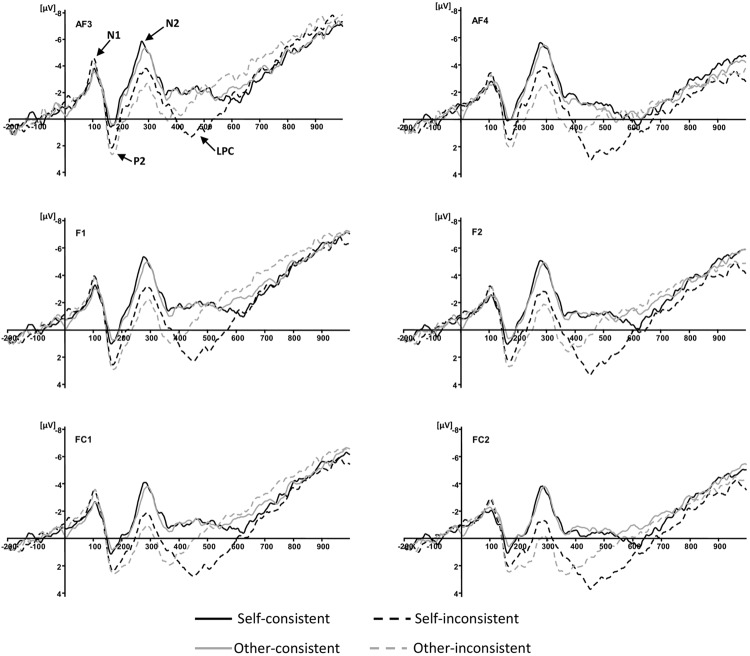
**Grand-averaged event-related potential waveforms elicited by four conditions at anterior electrode sites**.

**FIGURE 3:**
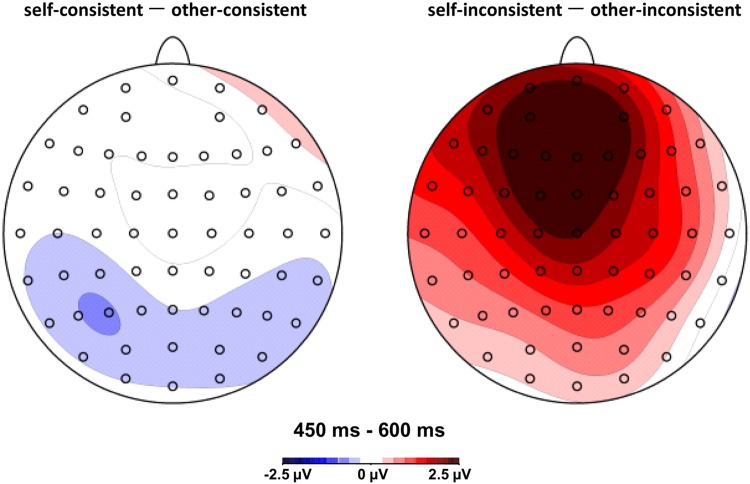
**Topographic maps of difference waves: other-consistent condition subtracted from self-consistent condition **(left** panel), and other-inconsistent condition subtracted from self-inconsistent condition **(right** panel) in the 450–600 ms post-stimulus epoch**.

According to previous studies (Sabbagh and Taylor, 2000; Liu et al., 2004, 2009a,b; Meinhardt et al., 2011; Bowman et al., 2012; Jiang et al., 2016), the components associated with ToM are normally over the frontal region. In particular, recent studies confirmed the important role of the pMPFC in the decoupling mechanism of distinguishing the mental state of others from one’s own in ToM reasoning (Schuwerk et al., 2014a,b). Visual inspection of the averaged waveforms of each condition and the topographic difference maps in the present study also suggested that the divergence of waveforms elicited by the four conditions distributed around frontal and fronto-central areas (see **Figures 2** and **3**). For these reasons, five pairs of electrodes including AF3/AF4, F1/F2, F3/F4, FC1/FC2, and FC3/FC4, were selected for subsequent statistical analysis. As shown in **Figure 2**, four ERP components were elicited by time-locked stimulus including N1 (70–150 ms), P2 (120–200 ms), N2 (200–400 ms), and LPC (400–600 ms). The amplitude of N1, P2, N2, and 50 ms interval mean amplitudes of LPC, were analyzed with a 5 × 2 × 2 × 2 repeated-measures analysis of variance (ANOVA) with electrode pairs (AF3/AF4, F1/F2, F3/F4, FC1/FC2, and FC3/FC4), hemisphere (left vs. right), agency (self vs. other), and consistency (consistent vs. inconsistent). To correct for the capitalization, only if at least three consecutive significant intervals (*p* < 0.05) of 50 ms were accepted as truly significant and reported (Gomarus et al., 2009). *P*-values were corrected using the Greenhouse-Geisser method where appropriate.

## Results

### Behavioral Results

The accuracy of the self-consistent, self-inconsistent, other-consistent, and other-inconsistent conditions was 97.4, 95.4, 96.6, and 90.6%, respectively. A 2 × 2 repeated-measures ANOVA with agency (self vs. other), and consistency (consistent vs. inconsistent) revealed a significant difference between the accuracy of self conditions (96.4%) and that of other conditions (93.6%), *F*(1,15) = 6.62, *p* = 0.02, $\eta_{p}^{2}$ = 0.31. There was also a significant difference between the accuracy of consistent conditions (97.0%) and that of inconsistent conditions (93.0%), *F*(1,15) = 17.41, *p* = 0.001, $\eta_{p}^{2}$ = 0.54. The interaction between agency and consistency was also significant, *F*(1,15) = 5.63, *p* = 0.03, $\eta_{p}^{2}$ = 0.27. Simple effects analyses showed that the accuracy of self-inconsistent condition was greater than that of other-inconsistent condition, *F*(1,15) = 7.04, *p* = 0.02, $\eta_{p}^{2}$ = 0.32, whilst the difference between the accuracy of self-consistent condition and that of other-consistent condition was not significant.

### ERP Results

#### Late Positive Component (LPC)

During the LPC (400–600 ms) time window, the overall analysis showed that the interaction between agency and consistency was consecutively significant for the 450–500, 500–550, and 550–600 ms post-stimulus epochs, *F*(1,15) = 21.51, *p* < 0.001, $\eta_{p}^{2}$ = 0.59, *F*(1,15) = 26.24, *p* < 0.001, $\eta_{p}^{2}$ = 0.64, and *F*(1,15) = 10.79, *p* = 0.005, $\eta_{p}^{2}$ = 0.42, respectively. Simple effects analyses showed that for 450–500 ms, the belief reasoning for self (*M* = 2.10, *SD* = 0.85) elicited a more positive waveform than the belief reasoning for others (*M* = -0.31, *SD* = 1.06) when a participant’s own belief was inconsistent with others, *F*(1,15) = 16.13, *p* = 0.001, $\eta_{p}^{2}$ = 0.52; for 500–550 ms, the belief reasoning for self (*M* = 1.55, *SD* = 1.09) also had a more positive waveform than the belief reasoning for others (*M* = -0.91, *SD* = 1.16) when a participant’s own belief was inconsistent with others, *F*(1,15) = 23.93, *p* < 0.001, $\eta_{p}^{2}$ = 0.62; for 550–600 ms, the belief reasoning for self (*M* = 0.61, *SD* = 1.13) continued to elicit a more positive waveform than the belief reasoning for others (*M* = -1.52, *SD* = 1.28) when a participant’s own belief was inconsistent with others, *F*(1,15) = 14.97, *p* = 0.002, $\eta_{p}^{2}$ = 0.50. However, there was no significant difference between belief reasoning for self and for others when beliefs were consistent for the three consecutive epochs. The results have been further confirmed by the topographic difference maps shown in **Figure 3**.

#### N1, P2, and N2

N1: No experimental main effect or interaction was significant for the N1 amplitude.

P2: The main effect of consistency for the P2 amplitude was significant, *F*(1,15) = 12.19, *p* = 0.003, $\eta_{p}^{2}$ = 0.45, with a more positive P2 for inconsistent conditions (*M* = 4.07, *SD* = 0.61) than for consistent conditions (*M* = 2.79, *SD* = 0.59).

N2: The main effect of consistency for the N2 amplitude was significant, *F*(1,15) = 7.65, *p* = 0.01, $\eta_{p}^{2}$ = 0.34, with a greater negativity for consistent conditions (*M* = -5.83, *SD* = 1.15) than for inconsistent conditions (*M* = -3.92, *SD* = 0.86).

## Discussion

To address the electrophysiological bases underlying the decoupling mechanism in ToM processing, the current study compared the time course of neural activities associated with belief reasoning for self and for other people under consistent and inconsistent conditions. The behavioral results revealed that the accuracy of belief reasoning for self was significantly greater than that of belief reasoning for others when the participant’s own belief was inconsistent with others. However, the difference between belief reasoning for self and for others was not significant when the participant and other people shared the same belief. These findings suggest that belief reasoning for self and for others is equivalent in processing difficulty when beliefs belonging to different agencies are the same. While belief reasoning for self could be easier and faster than that for others if beliefs are in conflict with each other. The behavioral finding is in line with the ERP results of LPC in the present study, which also revealed a significant interaction between agency and consistency and confirmed the difference between belief reasoning for self and for others in inconsistent conditions.

The present study revealed that the electrophysiological divergence was most clearly pronounced on the LPC with an anterior scalp distribution. The findings are consistent with prior ERP studies concerning ToM respect to latency and topography (Sabbagh and Taylor, 2000; Liu et al., 2004, 2009a,b; Bowman et al., 2012; Jiang et al., 2016). The most intriguing finding in the present study was the significant interaction between agency and consistency on the amplitudes of LPC during the 450–600 ms epoch. Results revealed that during the three consecutive epochs, 450–500, 500–550, and 550–600 ms, the mean amplitude of belief reasoning for self was more positive than that of belief reasoning for others when the beliefs belonging to different agencies were inconsistent with each other. There was no difference between the mean amplitude of belief reasoning for self and for others when beliefs were consistent during the same epochs. Interestingly, our prior study concerning another type of mental state in ToM, desire reasoning, obtained a similar pattern of results (Jiang et al., 2016). In that study, we investigated the neural bases of desire reasoning for self and for others when desires were either consistent or inconsistent. The interaction between agency and consistency of LPC was also observed. The LPC associated with desire reasoning was larger in the condition of reasoning for self than that for others when desires were inconsistent. Since both the current study concerning belief and the study concerning desire show the effect of LPC divergence between mental state reasoning for self and for others, it suggests that the LPC is responsive to a common substrate involved in ToM processes.

An important substrate involved in ToM processes is the decoupling mechanism distinguishing another’s mental state independently from one’s own (Gallagher and Frith, 2003). Additionally, Frith and Frith (1999) proposed that the substrates of ToM included the ability to distinguish actions between one’s own and those of others. Given the time windows and the anterior scalp distribution, the LPC in the present study showed similarity with the P3 component which is associated with stimuli categorization and evaluation (Ito and Cacioppo, 2000; Batty and Taylor, 2002; Polich, 2007; Hu et al., 2011). Azizian et al. (2006) investigated the electrophysiological correlates of categorizing stimuli varying in similarity and found a larger P3 for stimuli similar to target than other stimuli. The authors argued that the P3 amplitude may reflect the categorization based on similarity. Moreover, Chen et al. (2006) reported that the judgment of category was manifested on LPC. They proposed that LPC is responsible for high-level categorization. In the present study, the target event to which the ERP data were time-locked was the question asking participants to predict different agencies’ behavior. Since the rest part of the question already presented in the previous screen, the only important target which participants needed to identify was the word of agency: “you” for self conditions, and “he/she” for other conditions. To provide a correct response to the question, participants needed to consider the belief of the agency mentioned in the question and then inferred a behavior based on that belief. In inconsistent conditions, participants’ own belief about the location of the ball was in conflict with others, which required participants to first categorize agencies to whom the belief was attributed. There was no need to categorize different agencies since both they and other people shared a belief about the location of the ball in consistent conditions. Thus, the dissociation of LPC occurring in mental state reasoning for self and for others may represent the categorization of agencies to which mental states are attributed in ToM processes. Although previous studies have not discussed the ERP difference between reasoning for self and for others in ToM directly, results in the present study can be compared with ERP studies concerning the processing of information about self and other. Many studies reported the self-preference effect on P3, which showed that processing for self is associated with higher P3 amplitude than for others (Hu et al., 2011; Tacikowski et al., 2014; Kotlewska and Nowicka, 2015). It is noteworthy that the self-related information differs from other-related information in these studies. Consistent with these findings, the present results revealed that a more positive waveform was elicited by belief reasoning for self than for others when beliefs belonging to self and others were different in the 450–600 ms epoch, which can be associated with the categorization of different agencies involved in the decoupling mechanism as necessary.

With respect to spatial distribution, the interaction effect was most clearly observed in anterior regions (see **Figure 3**). There was no hemisphere lateralization effect including the main factor of agency for belief reasoning observed in the present study. It is consistent with a previous fMRI study by Saxe et al. (2006), which reported that both belief reasoning for others and self-reflection activated the medial prefrontal and medial precuneus regions with no lateral effect. It is also supported by recent findings of Schuwerk et al. (2014a,b): evidence from both fMRI and rTMS studies suggest that the pMPFC plays a key role in the decoupling mechanism to distinguish the self from the others in ToM reasoning. However, until now, the hemisphere lateralization effect is still controversial in light of the task diversity in different studies (Sabbagh and Taylor, 2000; Liu et al., 2004, 2009a,b; Zhang et al., 2009; Bowman et al., 2012). Further studies are needed to confirm the validity and meaning of the hemisphere effect in mental states reasoning.

Although the assumption of categorization for different agencies in the decoupling mechanism is supported by the aforementioned findings, an alternative explanation for the dissociation of LPC should be considered. That is the different inhibiting demands loaded in belief reasoning for self and for others when beliefs are conflicting might account for the divergence of LPC observed in the current study. The present findings cannot eliminate the possibility since the LPC over the anterior area is also proposed to be associated with conflict resolution and inhibitory control (Zhang et al., 2009). Further research is required to examine the validity of the two explanations.

The results also showed the differences of early components, including P2 and N2. All of them were associated with the effect of consistency. According to the experimental procedure used in the current study, participants gained the information of the beliefs belonging to themselves which were consistent or inconsistent with others before the presentation of the question as a target event. Thus, participants may choose different strategies to allocate cognitive resources for early processing in consistent and inconsistent conditions, which result in the effects of early components. The cognitive processing reflected by the early components, such as attention and perception (Mangun, 1995; Bigman and Pratt, 2004), provides some preparation for subsequent ToM reasoning. However, considering that the components associated with ToM are normally later than 300 ms after onset of stimulus (Sabbagh and Taylor, 2000; Liu et al., 2004, 2009a,b; Meinhardt et al., 2011, 2012; Bowman et al., 2012; Kühn-Popp et al., 2013; Geangu et al., 2013), the effects of early components are unlikely to be informative about ToM reasoning *per se* (McCleery et al., 2011). These results may suggest that the present study design is not appropriate to investigate early components in a reliable manner. We will try to improve the experimental design in the future studies concerning ToM reasoning to avoid the confusion about early components.

## Conclusion

The present study shed first light on the neural correlates of belief reasoning for self and for others under consistent and inconsistent conditions. Results found that during the 450–600 ms time period, the LPC elicited by belief reasoning for self was more positive than for others when beliefs were inconsistent with each other, which is assumed to represent the categorization of different agencies in ToM processes.

## Author Contributions

QJ: Study design, data collection, data analyzing, paper writing. QW: Data analyzing, paper writing. PL: Data analyzing, paper writing. HL: Study supervision, theoretical discussion.

## Conflict of Interest Statement

The authors declare that the research was conducted in the absence of any commercial or financial relationships that could be construed as a potential conflict of interest.
